# Acrylamide Mitigation in Popcorn: A Comparison of Innovative Techniques

**DOI:** 10.3390/foods15122049

**Published:** 2026-06-06

**Authors:** Albert Sebastià, Carmen Fernández-Matarredona, Francisco J. Barba, Houda Berrada, Olga Pardo, Francesc A. Esteve-Turrillas, Emilia Ferrer, Pedro V. Martínez-Culebras

**Affiliations:** 1 Research Group in Innovative Technologies for Sustainable Food (ALISOST), Preventive Medicine and Public Health, Food Science, Toxicology and Forensic Medicine Department, Faculty of Pharmacy and Food Sciences, University of Valencia, Av. Vicent Andrés Estellés, 22, 46100 Burjassot, Spain; carferm2@alumni.uv.es (C.F.-M.); francisco.barba@uv.es (F.J.B.); emilia.ferrer@uv.es (E.F.); pedro.martinez@uv.es (P.V.M.-C.); 2 CIBER de Enfermedades Infecciosas (CIBERINFEC), Instituto de Salud Carlos III, C/Monforte de Lemos 3-5. Pabellón 11, Planta 0, 28029 Madrid, Spain; 3 Research Group in Alternative Methods for Determining TOXICS Effects and Risk Assessment of Contaminants and Mixtures RiskTox Laboratory of Food Chemistry and Toxicology, Faculty of Pharmacy and Food Sciences, University of Valencia, Av. Vicent Andrés Estellés s/n, 46100 Valencia, Spain; 4 Department of Analytical Chemistry, University of Valencia, 50th Dr. Moliner St., 46100 Burjassot, Spain; olga.pardo@uv.es (O.P.); francesc.a.esteve@uv.es (F.A.E.-T.)

**Keywords:** acrylamide, popcorn, soaking, PEF, ultrasound, cereal processing

## Abstract

Acrylamide (AA), a food processing contaminant and potential carcinogen, poses a significant health risk in heat-processed snacks, particularly for children. This study evaluates the efficacy of three pre-treatments: pulsed electric fields (PEFs), ultrasound (USN), and soaking for AA mitigation in popcorn (*Zea mays everta*). Using liquid chromatography–tandem mass spectrometry (LC-MS/MS), AA levels were quantified across nine treatment variations. All strategies significantly reduced AA formation (*p* < 0.0001), with soaking (20 min) and USN (20 min) achieving the highest reductions (>82% and 82%, respectively). High-intensity PEF (3 kV cm^−1^, 300 kJ kg^−1^) yielded a 71% reduction, though it showed lower reproducibility due to the kernel’s dense morphology. Crucially, while soaking and USN were superior in AA leaching, durations exceeding 20 min compromised popping expansion and sensory texture due to excessive hydration. These results define the critical processing window for industry, balancing toxicological safety with product quality.

## 1. Introduction

In recent years, there has been growing concern about food safety in relation to acrylamide (AA), a potential carcinogen found in foods such as bread, fried potatoes, coffee, and popcorn [1,2]. AA forms during the Maillard reaction, a chemical process between reducing sugars and asparagine at temperatures above 120 °C, contributing to the flavor and the characteristic browning appearance of carbohydrate-rich foods [3,4].

Classified by the International Agency for Research on Cancer (IARC) as “probably carcinogenic to humans” (2A), AA exposure has also been linked to neurotoxicity and reproductive toxicity [5]. While definitive results in humans are still lacking, the European Food Safety Authority (EFSA) has raised concerns about its impact on public health [6,7]. AA is not genotoxic on its own, but it is metabolized to its epoxide form, glycidamide. This metabolite can cause DNA damage by forming covalent bonds, a process that can lead to fixed mutations and, ultimately, neoplastic transformation.

Despite these concerns, AA levels in food remain unregulated. Although benchmark levels have been established by the European Commission (EC) for several food categories, such as French fries, potato chips, and cereals, they omit specific limits for popcorn and other corn-based snacks [8]. However, the EC recommendation of 2019 identifies cereal snacks, such as popcorn or extruded maize products, as key matrices for monitoring AA levels. This regulatory gap underscores the need for targeted research on AA levels in popcorn and the development of practical, scalable mitigation strategies [9].

Popcorn, derived from the *Zea mays everta* variety, is a globally consumed snack known for its convenience and sensory attributes [10]. Popcorn’s production involves high-temperature processes providing optimal conditions for AA formation. This concern is amplified by the growing consumption of popcorn, particularly microwave and ready-to-eat varieties, across Europe and globally, especially during the COVID-19 pandemic and post-pandemic periods, when snacking behaviors changed dramatically. The broad range of AA levels reported in popcorn (0.5–14,951 µg kg^−1^) underscores the variability in production methods and the need for better control and understanding of mitigation strategies [11,12].

Risk assessment studies on AA intake in the Spanish population based on popcorn consumption suggest that while average intake levels may fall below critical thresholds for adults, children (who often consume more snacks relative to their body weight) may be at elevated risk [11]. A previous study reported that the margin of exposure (MOE) for children fell below the safety threshold of 10,000 in pessimistic intake scenarios, raising concern among regulators and public health officials [11].

Despite the relevance of the issue, there is a notable lack of published studies addressing AA mitigation in maize (popcorn) or other cereals using innovative technologies. At an industrial scale, few mitigation measures have been reported for AA formation in cereal products. This is in sharp contrast to the potato industry, where conventional methods like aqueous soaking are widely used [13,14]. However, in recent years, innovative technologies such as pulsed electric fields (PEF) and ultrasound (USN) have emerged, demonstrating comparable mitigation effects [15,16]. In this context, well-designed experimental studies are urgently needed to study the effectiveness of soaking and emerging treatments in reducing AA levels in cereal matrices.

Innovative physical and technological interventions offer a promising alternative. For instance, PEF treatment involves applying short bursts of low-/medium-voltage electric fields to food matrices, which induces electroporation (disruption of cell membranes) either reversibly or irreversibly, depending on the intensity. In plant tissues like corn kernels, this increased membrane permeability enhances the release of intracellular compounds into the surrounding environment [17]. This permeabilization promotes the facilitated diffusion of precursors such as free asparagine and reducing sugars, which are of particular relevance to AA mitigation [18,19,20].

Free asparagine is often the limiting precursor in AA formation, and its availability strongly influences the extent of AA generated. PEF-induced cell membrane permeability facilitates the migration of asparagine from the grain’s interior to its surface or into an aqueous environment. As a result, less asparagine remains available to react with carbonyl groups from reducing sugars during high-temperature cooking, thereby reducing AA formation [21,22].

This mechanism has been extensively studied in food matrices such as potatoes and sweet potatoes, with AA reductions ranging from 17 to 31% reported following deep-frying. These effects are typically achieved under electric field strengths of around 1.5 kV cm^−1^ [21,23].

On the other hand, USN induces cavitation phenomena, promoting disruption of cell integrity, and may reduce AA precursors or improve their extractability before heat treatment. Moreover, USN can improve the efficacy of other mitigation strategies when applied in combination, such as enhancing the performance of soaking or soaking steps [24,25]. This technology has also been applied to fried potatoes, achieving AA reductions of up to 50% under conditions of 35 kHz, 92.5 W kg^−1^, and 42 °C [26].

Another traditional, but effective method, is soaking, typically involving immersing samples in hot water for a short time. This treatment can leach out reducing sugars and free asparagine. Although widely used in other starchy matrices such as potatoes, the application of soaking to popcorn kernels is not well documented in the literature, and its effect on popping yield and texture remains a research gap [27].

The three aforementioned methods facilitate the release of precursors from the food matrix, thereby mitigating AA formation during subsequent thermal processing. Several studies have demonstrated a clear relationship between the application of these pre-treatments, the leaching of precursors, and the consequent reduction in AA levels. For instance, Santiago-Mora et al. [28] demonstrated that PEF treatment reduced the content of reducing sugars by 19% and asparagine by 42% in Lamoka potato slices, leading to a 28.9% decrease in AA. Similar results were reported by Liu et al. [29] when subjecting sweet potato chips to USN, where both reducing sugar and AA contents were noticeably decreased. Finally, Zhang et al. [30] applied a blanching step at temperatures ranging from 65 to 85 °C for durations of 2 to 10 min, achieving maximum reductions of 64.2%, 49.8%, and 61.3% for reducing sugars, asparagine, and AA, respectively.

While AA mitigation is well-documented in potato-based matrices, popcorn presents a unique challenge due to its anatomical structure and the high-pressure dynamics required for its expansion. Current European guidelines omit specific limits for popcorn, despite its significant contribution to the dietary exposure of vulnerable populations. This study fills a critical research gap by comparatively evaluating innovative (PEF, USN) and traditional (soaking) technologies to mitigate AA. The findings contribute to the development of practical and sustainable mitigation approaches and offer valuable guidance for regulatory frameworks and industrial processing practices targeting AA reduction in cereal-based snacks.

## 2. Materials and Methods

### 2.1. Chemicals and Reagents

Analytical-grade AA and its deuterated analog (AA-d_3_), both with purity levels exceeding 99%, were procured in solid form from Merck (Darmstadt, Germany). These standards were utilized to prepare stock solutions at a concentration of 1 mg L^−1^ using high-purity deionized water. For all experimental stages, deionized water (resistivity >18 MΩ cm^−1^) was generated through a Milli-Q SP Reagent Water System (Millipore Corporation, Bedford, MA, USA). Magnesium oxide (MgO), formic acid, and zinc sulfate heptahydrate (ZnSO_4_·7H_2_O) were sourced from Thermo Fisher Scientific (Madrid, Spain). The filtration of liquid extracts was carried out in two stages: an initial pass through VWR Grade 302 filter paper (8–12 μm, Darmstadt, Germany), followed by a final clarification step using 13 mm/0.22 μm nylon membranes from Membrane Solutions (Plano, TX, USA) prior to LC-MS/MS analysis.

### 2.2. Treatments

**Figure 1:**
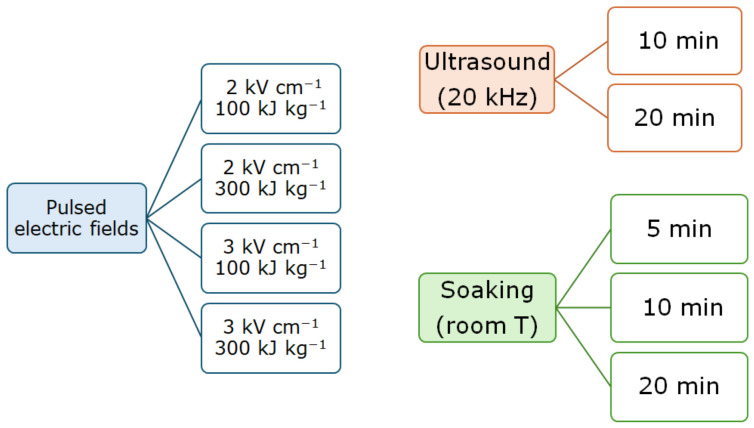
Overview of the different pre-treatments and conditions applied for acrylamide mitigation in popcorn.

Popcorn kernels were pre-treated using three different methods: PEF, USN, and soaking. Figure 1 provides a general overview of the parameters used for each treatment.

#### 2.2.1. Pulsed Electric Field Treatment

PEF processing was performed utilizing a PEF-Cellcrack III system (German Institute of Food Technologies (DIL); ELEA, Quakenbrück, Germany), equipped with a treatment chamber featuring a 10 cm electrode gap. For each trial, 65 g of maize kernels were immersed in 200 mL of Milli-Q water. To standardize the electrical conditions, the conductivity of the resulting suspension was calibrated to 900 µS cm^−1^ using sodium chloride. Although the process was initiated at ambient temperature, real-time monitoring confirmed that the final temperature did not exceed 65 °C, even under the most intensive energy conditions.

The experimental design involved voltage settings of 20 and 30 kV, which generated specific electric field strengths of 2 and 3 kV cm^−1^ and energy inputs of 100 and 300 kJ kg^−1^, respectively. The delivery of these fields consisted of 59 to 398 pulses, administered in sequential cycles of 50 pulses each. The total operational time for the PEF-based mitigation strategy was strictly maintained under 5 min to ensure process efficiency.

#### 2.2.2. Ultrasound Treatment

USN treatment was performed in an ultrasonic bath (A Branson 5200, Danbury, CT, USA); the conditions employed were 20 kHz frequency and 100 W power. The duration assessed was 10 and 20 min. The mass of the sample treated was also 65 g.

#### 2.2.3. Soaking Treatment

Soaking treatment was performed by immersing 65 g of corn kernels in 200 mL of Milli-Q water. The kernels were continuously stirred for 5, 10, and 20 min at room temperature.

### 2.3. Thermal Process

A total of 65 g of corn was cooked employing the Fun&Taste PCorn easy 200 W popcorn maker (Cecotec, Quart de Poblet, Valencia, Spain). The cooking process did not exceed 2 min.

### 2.4. Sample Preparation

Quantification of AA has been validated in a previous work [31]. The isolation of AA from popcorn matrices was achieved using a solid–liquid extraction (SLE) procedure, followed by liquid chromatography–tandem mass spectrometry (LC-MS/MS) analysis, in accordance with established protocols. Samples (65 g) were first pulverized to a fine consistency using a Pulverisette 11 mill (Fritsch, Madrid, Spain). For the extraction, a 1 g subsample of the homogenized powder was fortified with 333 µL of the internal standard (AA-d_3_, 1 mg L^−1^). Following a 15 min equilibration period, the sample was diluted with 10 mL of Milli-Q water. It was then processed in a Branson 5200 ultrasonic bath (20 kHz, 100 W), followed by 10 min of mechanical shaking using a MultiMix Heat D stirrer (Ovan, Barcelona, Spain). To separate the phases, the mixture underwent centrifugation at 2504× *g* for 10 min in an M-Universal centrifuge (MPW Med. Instruments, Warsaw, Poland). A 3.5 mL aliquot of the resulting supernatant was transferred to a 15 mL tube for a cleanup step involving 0.25 g of MgO and 0.25 g of ZnSO_4_·7H_2_O. After 1 min of vortexing (Vortex-Vib, J.P. SELECTA S.A., Barcelona, Spain) and a second centrifugation cycle (2504× *g* for 10 min), 1.5 mL of the clarified aqueous phase was collected. This volume was then reduced to dryness under a nitrogen stream using a TurboVap LV evaporator (Zymark Corp., Hopkinton, MA, USA). Finally, the dry residue was reconstituted in 0.5 mL of Milli-Q water, vortexed for 1 min, and centrifuged again (2504× *g*, 10 min) to remove any residual starch. The final extract was passed through a 0.22 µm nylon membrane before being injected into the LC-MS/MS system.

### 2.5. Acrylamide Determination

The AA quantification was conducted using an Agilent 1100 Series high-performance liquid chromatograph (Agilent Technologies, Palo Alto, CA, USA) coupled to a Finnigan TSQ Quantum Ultra triple quadrupole mass spectrometer (Thermo Fisher Scientific, Waltham, MA, USA). The chromatographic separation was achieved with an Aquasil C18 column (100 × 2.1 mm, 3 μm particle size; Thermo Scientific). A 5 μL volume of either the standard solution or the prepared sample extract was injected, and separation was performed in isocratic mode using 0.1% formic acid in Milli-Q water as the mobile phase, delivered at a flow rate of 0.2 mL min^−1^.

Mass spectrometric detection was carried out under multiple reaction monitoring (MRM) conditions using an atmospheric pressure chemical ionization (APCI) source in positive ion mode. Source parameters included a discharge current of 3 μA, a vaporizer temperature of 250 °C, and a capillary temperature of 350 °C. Nitrogen gas, supplied by a Zefiro 35 LC-MS generator (Cinel Gas Generators, Vigonza, Italy), was used as both the sheath gas (25 psi) and the auxiliary gas (3 psi) to aid in nebulization and desolvation. Argon was employed as the collision gas at a pressure of 1.0 mTorr.

MRM transitions monitored were *m*/*z* 72 > 44 and 72 > 55 for native AA, and *m*/*z* 75 > 44 and 72 > 58 for the internal standard (AA-d_3_). The collision energies applied were 24 V and 11 V for the respective transitions. Instrument control and data acquisition were managed using TSQ Series 2.3 SP3 software, while data processing was performed with Xcalibur software version 2.2 SP1.48 (Thermo Fisher Scientific).

### 2.6. Statistical Analysis

All treatments were performed in triplicate, and results were expressed as mean ± standard deviation. A one-way analysis of variance (ANOVA) was used to determine statistically significant differences between treatments, followed by Tukey’s post hoc test for multiple comparisons (*p* < 0.05). Statistical grouping was indicated using different superscript letters in figures. The percentage reduction in AA level was calculated by comparing each treated sample to the untreated control. GraphPad Prism (version 9.0.0; GraphPad Software Inc., San Diego, CA, USA) was used for data analysis and graphical representation.

## 3. Results

This study investigated the effect of various treatment methods on AA levels in popcorn samples. A total of nine different treatment conditions were analyzed, including PEF at four intensities (2 kV cm^−1^ and 3 kV cm^−1^, both at 100 and 300 kJ kg^−1^), USN (at 10 and 20 min), and soaking (at 5, 10, and 20 min).

### 3.1. Effect of Pulsed Electric Fields Treatments

**Figure 2:**
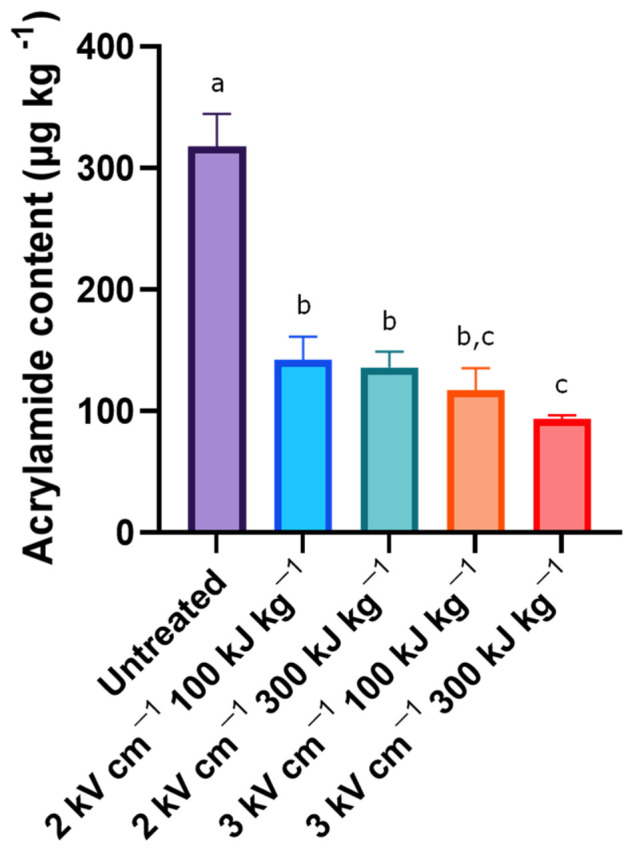
Acrylamide levels in popcorn samples subjected to different pulsed electric field treatments. Values are expressed as mean ± standard deviation. Bars not sharing a common letter are significantly different (*p* < 0.05).

As shown in Figure 2, PEF treatments significantly reduced AA levels compared to untreated controls (*p* < 0.0001). The most effective configuration was 3 kV cm^−1^ at 300 kJ kg^−1^, which resulted in an average AA reduction of 71% (Figure 2). However, no statistically significant differences were observed among the other PEF treatments (2 kV cm^−1^ at 100 and 300 kJ kg^−1^, and 3 kV cm^−1^ at 100 kJ kg^−1^), which all yielded roughly a 60% reduction. This suggests a plateau effect at lower energy levels and field strengths.

### 3.2. Effect of Ultrasound Treatments

**Figure 3:**
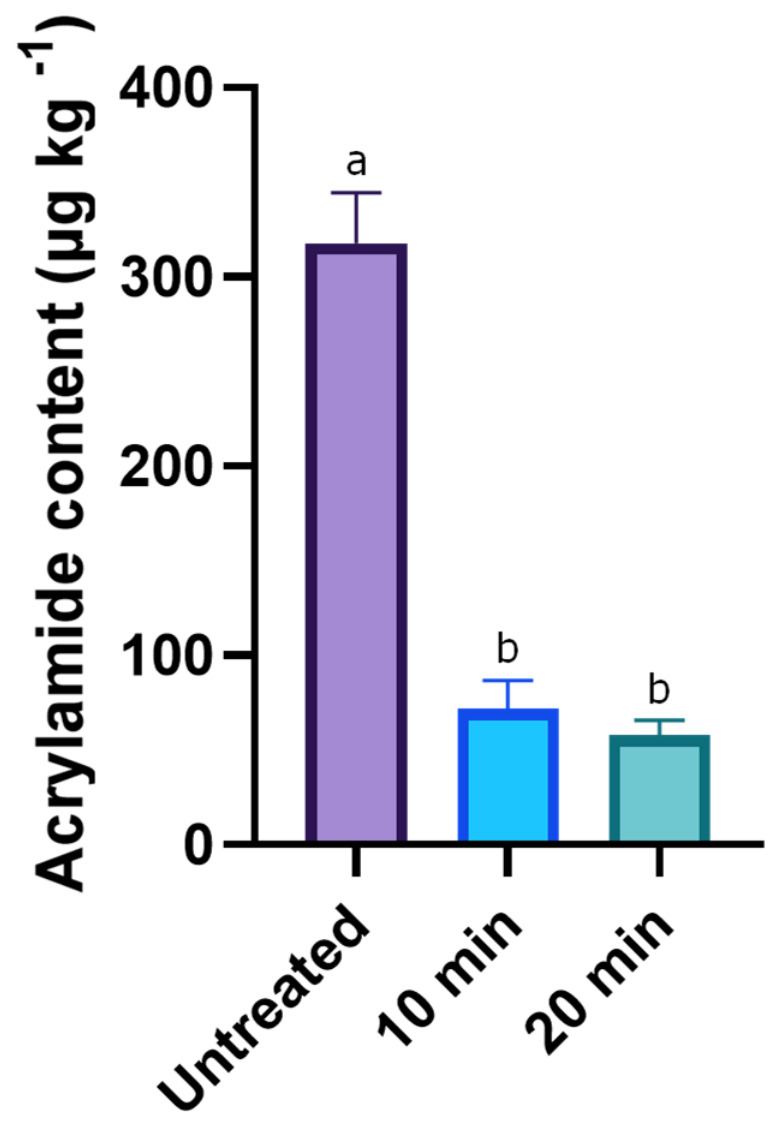
Acrylamide levels in popcorn samples subjected to different ultrasound treatments. Values are expressed as mean ± standard deviation. Bars not sharing a common letter are significantly different (*p* < 0.05).

USN treatments also resulted in substantial AA reductions. The 10 min USN treatment achieved a 77% reduction, while the 20 min treatment reached 82%. Both treatments showed statistically significant reductions compared to the control (*p* < 0.0001), although there were no significant differences between them, indicating diminishing returns beyond 10 min (Figure 3).

### 3.3. Effect of Soaking Treatments

**Figure 4:**
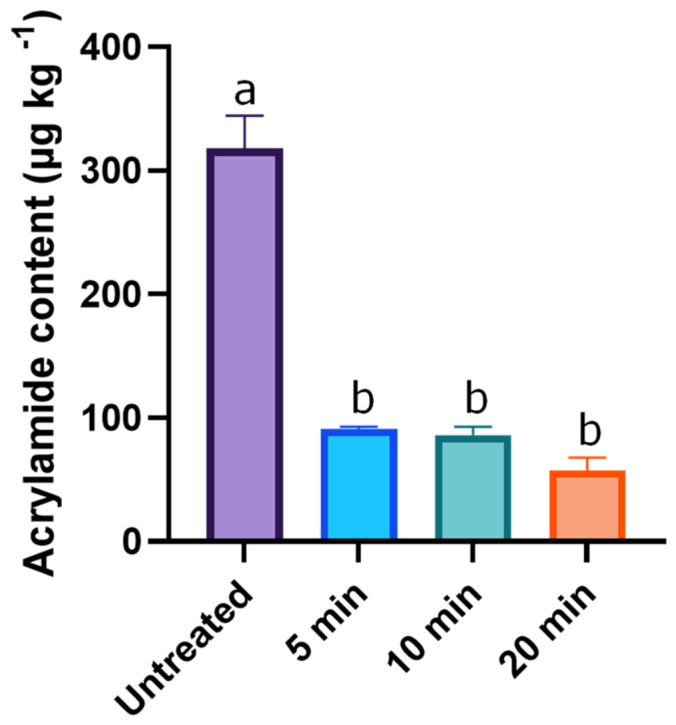
Acrylamide levels in popcorn samples subjected to soaking for 5, 10, and 20 min. Values are expressed as mean ± standard deviation. Bars not sharing a common letter are significantly different (*p* < 0.05).

Soaking at 25 °C for 5, 10, and 20 min progressively reduced AA levels. A 5 min treatment achieved 71% reduction (91 µg kg^−1^), whereas the 20 min treatment reached the lowest AA level (58 µg kg^−1^), corresponding to a reduction of over 82%. All soaking durations resulted in statistically significant reductions compared to the control (*p* < 0.0001); however, no significant differences were observed among the different soaking times (*p* > 0.05), suggesting an early saturation effect in the leaching of AA precursors (Figure 4).

### 3.4. Overall Comparison of Treatments

**Figure 5:**
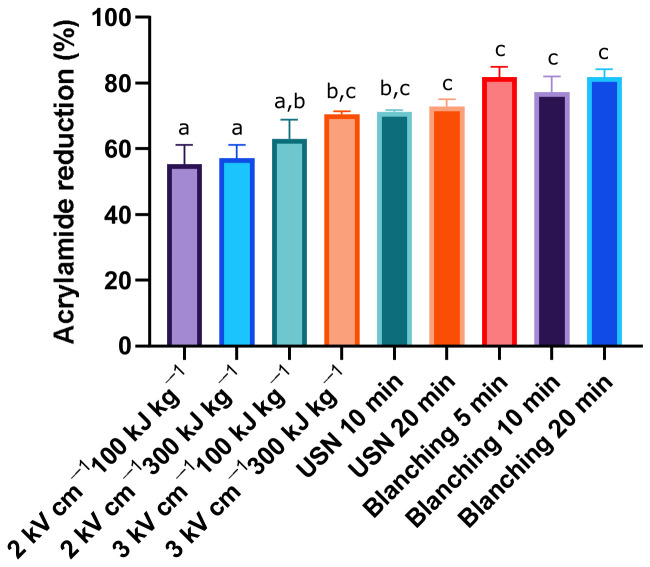
Acrylamide reduction (%) in popcorn samples subjected to different mitigation treatments. Values represent mean ± standard deviation. Treatments not sharing a common letter differ significantly (*p* < 0.05). PEFs: pulsed electric fields; USN: ultrasound.

Figure 5 presents a comprehensive comparison of AA reduction percentages for all pre-treatments. Soaking for 20 min achieved the highest mitigation (>82%), followed closely by USN for 20 min. USN and soaking consistently outperformed all PEF conditions. Among the PEF treatments, 3 kV cm^−1^ at 300 kJ kg^−1^ was the most effective (71%), while the lower-intensity treatments hovered near 60%. The graph highlights a hierarchy of efficacy: soaking ≥ USN > PEF.

## 4. Discussion

This study confirms that pre-treatment strategies play a crucial role in mitigating AA formation in popcorn. Among the tested interventions, soaking, especially for 20 min, achieved the most significant reduction, lowering AA levels by over 80% and well below the proposed benchmark of 150 µg kg^−1^. These results support existing evidence that water-based treatments are effective in leaching out key precursors such as free asparagine and reducing sugars prior to thermal processing.

USN also showed promising results; the cavitation effect likely enhanced precursor removal or induced structural changes that interfered with AA formation. Although slightly less effective than soaking, USN may be advantageous in scenarios where thermal soaking is impractical or undesirable.

In contrast, PEF demonstrated more modest reductions. While high-intensity settings (3 kV cm^−1^, 300 kJ kg^−1^) achieved up to 71% reduction, lower-intensity conditions were significantly less effective. This could be due to the dense and impermeable structure of popcorn kernels, which limits the mass transfer and electroporation effects typically exploited by PEF. Furthermore, the variability in AA levels observed in PEF-treated samples indicates lower reproducibility and control (coefficient of variation (CV) = 9–11%) compared to both soaking (CV = 1–4%) and USN (CV = 3–6%).

**Figure 6:**
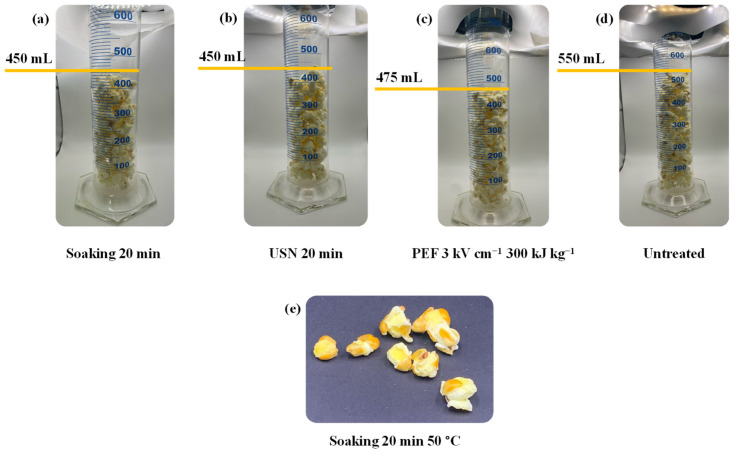
Qualitative comparison of popcorn expansion volume. Samples were either untreated (**d**), treated at 25 °C with (**a**) soaking (20 min), (**b**) ultrasound (20 min), or (**c**) pulsed electric fields (3 kV cm^−1^, 300 kJ kg^−1^). The figure also includes popcorn treated by (**e**) soaking for 20 min at 50 °C.

An important practical limitation related to the expansion volume of the popcorn was observed for all three technologies. To comparatively evaluate this phenomenon, 10 g of popcorn from each of the most intense treatments were weighed, and their volume was measured using a 1 L graduated cylinder. As shown in Figure 6, the untreated control reached a volume of approximately 500 mL, whereas the volume for the other treatments remained below 450 mL.

This effect was exacerbated when treatments were combined with temperatures above 25 °C. Soaking times longer than 20 min or using water temperatures above room temperature (25 °C) resulted in undesirable changes in popcorn quality, as can be observed in Figure 6e. Specifically, these conditions led to a decrease in expansion volume and compromised organoleptic properties such as texture and crunchiness. These effects are likely due to excess water absorption, which alters the internal pressure dynamics required for proper popping. Therefore, while soaking is highly effective for AA reduction, its operational parameters must be carefully optimized to avoid deteriorating product quality.

The observed hierarchy of efficacy (soaking > USN > PEF) highlights the role of mass transfer in whole-kernel systems. The relative underperformance of PEF (max 71% reduction) compared to soaking (>82%) can be attributed to the popcorn kernel’s pericarp. Unlike the parenchymatous tissue of potatoes, the dense and lignified structure of *Zea mays everta* likely limits the electroporation effect, hindering the outward diffusion of free asparagine into the medium [32]. Conversely, USN-induced cavitation and thermal leaching during soaking appear to be more effective at penetrating or softening this barrier, facilitating a more consistent extraction of AA precursors before thermal processing.

Other strategies to reduce AA levels in food include the use of low-asparagine raw materials, enzymatic treatments such as asparaginase, lactic acid bacteria fermentation, pH adjustment, and thermal process optimization. These approaches primarily aim to limit the availability of AA precursors or to alter reaction conditions. Among these, enzymatic and fermentation-based methods show particularly promising results in cereal-based products, often without compromising sensory attributes [33]. However, their application in whole-kernel systems like popcorn remains challenging due to structural limitations.

From a regulatory and industrial perspective, soaking stands out as a cost-effective, scalable, and simple intervention. The substantial reductions in AA levels observed in this study, coupled with its ease of implementation, position soaking as a viable strategy to improve the safety of popcorn products. Nevertheless, practical limitations such as impacts on expansion and texture must be considered to ensure consumer acceptability and consistent product performance. Table 1 summarizes the comparative effectiveness of the tested strategies by balancing their acrylamide-reduction potential against the resulting impacts on popping expansion and sensory attributes to identify the most viable industrial applications.

## 5. Conclusions

This study provides valuable insights for the AA mitigation toolbox by demonstrating the effectiveness of pre-treatment strategies in popcorn. However, selecting the optimal process requires balancing maximum AA reduction with the preservation of popping expansion and sensory attributes. While traditional soaking (20 min at 25 °C) achieved the highest AA reduction (>82%), it significantly compromised the popping volume and texture due to excessive water absorption, limiting its direct industrial application without further optimization. Therefore, the optimal recommended scheme for industrial application could be USN treatment for 20 min. This method offers the highest industrial potential by achieving an 82% reduction in AA levels while only moderately affecting the expansion quality of the popcorn. Alternatively, for processing lines where maintaining the original expansion volume and consistency is the primary priority, the optimal scheme is the application of PEF at 3 kV cm^−1^ and 300 kJ kg^−1^, which guarantees a substantial 71% AA reduction without altering the physical quality or texture of the final product. These clearly defined parameters offer practical, scalable, and science-based guidelines for the snack industry to effectively enhance food safety.

## Figures and Tables

**Table 1:** Integrated comparison of pre-treatment efficacy and its impact on popcorn quality.

Treatment	AA Reduction (%)	Expansion Quality	Industrial Approach
Soaking at 25 °C (20 min)	>82%	Significantly Reduced	Only with optimization
USN (20 min)	82%	Moderately Affected	Yes (High Potential)
PEF (3 kV cm^−1^ 300 kJ kg^−1^)	71%	Maintained	Yes (For consistency)

AA: acrylamide; USN: ultrasound; PEF: pulsed electric fields.

## Data Availability

The original contributions presented in this study are included in the article. Further inquiries can be directed to the corresponding authors.

## Abbreviations

The following abbreviations are used in this manuscript:

AA	Acrylamide
AA-d_3_	Deuterated Acrylamide
APCI	Atmospheric Pressure Chemical Ionization
EC	European Commission
EFSA	European Food Safety Authority
IARC	International Agency for Research on Cancer
LC-MS/MS	Liquid Chromatography–Tandem Mass Spectrometry
MOE	Margin of Exposure
MRM	Multiple Reaction Monitoring
PEF	Pulsed Electric Field
SLE	Solid–Liquid Extraction
USN	Ultrasound
